# Biobetters From an Integrated Computational/Experimental Approach

**DOI:** 10.1016/j.csbj.2017.01.003

**Published:** 2017-01-16

**Authors:** Serdar Kuyucak, Veysel Kayser

**Affiliations:** a School of Physics, University of Sydney, NSW 2006, Australia; b Faculty of Pharmacy, University of Sydney, NSW 2006, Australia

**Keywords:** Rational drug design, Molecular dynamics, Docking, Potential of mean force, Free energy perturbation

## Abstract

Biobetters are new drugs designed from existing peptide or protein-based therapeutics by improving their properties such as affinity and selectivity for the target epitope, and stability against degradation. Computational methods can play a key role in such design problems—by predicting the changes that are most likely to succeed, they can drastically reduce the number of experiments to be performed. Here we discuss the computational and experimental methods commonly used in drug design problems, focusing on the inverse relationship between the two, namely, the more accurate the computational predictions means the less experimental effort is needed for testing. Examples discussed include efforts to design selective analogs from toxin peptides targeting ion channels for treatment of autoimmune diseases and monoclonal antibodies which are the fastest growing class of therapeutic agents particularly for cancers and autoimmune diseases.

## 1 Introduction

Most of the drug leads that have high affinity for the target receptor ultimately fail because of problems with side effects, cytotoxicity or degradation. In fact, such problems are present in existing drugs but at a tolerable level. Improving the properties of existing biologics (protein or peptide-based drugs or drug leads) against such shortcomings is dubbed biobetters. Because the chemical space is very large, design of biobetters through trial and error methods is unlikely to succeed. One needs to make use of all the available information about the problems faced by a drug in order to facilitate the design of a biobetter. In fact, the experimental effort will be inversely proportional to the amount and accuracy of the information provided. As an example, consider solving the selectivity problem of a peptide ligand which binds to an off-target protein with a high affinity. If no information is available, one has to examine various mutations on the ligand which could be a very large experimental undertaking, e.g., for an average ligand with 30 amino acids, there are 30 × 19 = 570 single mutations and (30 × 29/2)× 19^2^ = 157,035 double mutations to consider. Using a docking program, one could identify the binding region on the ligand, which will reduce the number of mutations, e.g., if there are 4 residues in the hot spot, the number of single and double mutations will be reduced to 76 and 2166, respectively. While this is a drastic reduction, the experimental effort required is still substantial. As a next step, one could refine the binding poses obtained from docking using molecular dynamics (MD) simulations and obtain an accurate structure for the protein-ligand complex. Now one has a precise map of the inter-molecular interactions and can predict with some certainty which single and double mutations will yield the best outcome for reducing the affinity of the ligand for the off-target protein.

As illustrated in the above example, obtaining an accurate model of the protein-ligand complex holds the key for designing biobetters with minimal experimental effort. The most common method used for complex structure prediction is docking, which is fast but not very accurate. On the other extreme is MD, which can provide the desired accuracy but it is very slow. Combining the two methods by refining the binding poses obtained from docking in MD simulations offers a compromise solution that has been successfully applied to numerous protein-ligand complexes in the past decade [1], [2], [3]. An important ingredient in the success of this approach is the judicious use of the available experimental information about the complex system in the computations from initial docking to final validation. For example, available mutation data can be used as restraints in docking, which facilitates sampling of the correct pose and reduces the amount of subsequent MD work. Final validation of a predicted complex structure is typically based on binding free energy and available mutation data. While mutation of the residues in the predicted binding mode provides the most detailed and hence the best test for the proposed model, such data are not routinely available. Thus one may have to rely on the binding free energy of the ligand for validation, which has to be calculated near chemical accuracy to be useful for testing. Various methods can be used in calculation binding free energies from scoring functions in docking to potential of mean force (PMF) calculations in MD simulations. Again only the PMF calculations based on MD have the potential to provide the desired chemical accuracy.

Determination of validated complex structures is the most important step in design of biobetters because inspection of the binding mode will readily indicate the most promising mutations to achieve the desired improvement in affinity or selectivity. In fact, one can go beyond that and turn qualitative predictions into quantitative ones by calculating the effect of the mutation on the binding free energy from MD simulations. Such computational mutagenesis studies have the potential to eliminate guesswork completely and deliver the optimal biobetter for a given target with minimal side effects. In the following, we review the computational and experimental methods that will help to optimize design of biobetters while reducing the experimental efforts. Applications discussed include construction of selective analogs from toxin peptides targeting ion channels and design of biobetters from monoclonal antibodies with improved affinity and aggregation resistance.

## 2 Computational Methods

### 2.1 Protein-Ligand Complex Structure from Docking and MD

Determination of crystal structures for protein-ligand complexes is extremely difficult and very rare. Therefore, construction of an accurate complex structure from a given pair of protein and ligand structures is the most critical step in the design of a biobetter. Here we stress accuracy of the complex model in particular because an incorrect binding mode will predict misleading mutation sites for improvements, resulting in wasted experimental effort. Assuming crystal or NMR structures (or good homology models) of the protein and ligand are available, one can use a docking program to find a set of initial poses for the complex [4], [5]. Docking programs work by evaluating an energy function for various positions, orientations and conformations of the ligand with respect to the protein and ranking the energy scores. An energy function consists of Coulomb, van der Waals, and hydrophobic interactions and may include entropic terms. There are many commercial and academic docking programs, and choosing an appropriate one could be overwhelming. Most of them are for docking small drug-like molecules and would not be very useful for peptide ligands. Among the academic programs we mention AUTODOCK [6], [7], ZDOCK [8], and HADDOCK [9], [10]. AUTODOCK is the most popular docking program but works mainly for small molecules. ZDOCK can handle larger molecules like peptides but performs only rigid docking. Among the three, HADDOCK is most suitable for docking of peptide ligands as it can handle peptides and allows flexibility.

Accuracy of docking programs is limited due to neglect of water molecules and lack of adequate sampling [11]. These are automatically incorporated in MD simulations, hence MD has the capacity to provide an accurate representation of the protein-ligand interactions. However, MD is too slow to predict the complex structure from scratch. A compromise solution is to refine the binding poses predicted by docking in MD simulations, which avoids the shortcomings of either method and could provide the sought accuracy. This approach was first used for binding of small ligands (< 50 at.), and promising results were obtained [1], [12], [13], [14]. Feasibility of its extension to peptide ligands was initially demonstrated for binding of charybdotoxin to a KcsA potassium channel mimic using HADDOCK for docking [15], which was generalized to binding of other scorpion toxins to Kv channels in a subsequent systematic study [16]. For most channel-toxin complexes, a consensus complex was obtained from cluster analysis of the top 100 poses, which simplifies the refinement process with MD.

Several programs are available for performing MD simulations such as AMBER, CHARMM, GROMACS, and NAMD. The NAMD program [17] has been a popular choice because of its user-friendliness and the accompanying visualization and analysis software VMD [18]. Although NAMD allows use of different force fields, CHARMM has been the preferred choice in most simulations of proteins [19]. For the basic formalism of MD simulations, we refer to the monographs [20], [21]. Applications of MD simulations to membrane proteins, where creation of the simulation system is more involved, can be found in the reviews [22], [23], [24]. A key step in the refinement of the chosen binding pose via MD is the relaxation process where restraints between the protein and ligand are gradually reduced. The complex system is unlikely to be properly hydrated initially so without proper relaxation, various bonds and interactions in the complex may break, resulting in a dissociated ligand. There are well-established protocols for this purpose that can also be adapted for complex structures [25]. After relaxation, MD simulations are performed on the system, monitoring RMSDs of the protein and ligand, and the distances between interacting residues. The complex system is assumed to be equilibrated when the RMSDs reach a plateau and the time series of distances between interacting pairs fluctuate around a base line.

In the final stage, trajectory data obtained from the equilibrated system are used for visualization of the complex structure and analysis of the binding mode. The binding mode can be characterized quantitatively by calculating the average distances between the interacting residues. The strong ones include charge interactions, where the N—O distance between the charged residues is about 3 Å, and hydrophobic interactions involving aromatic side chains (2–3 kcal/mol). Intermediate strength interactions include hydrogen bonds and charge interactions at larger distances (1–2 kcal/mol). The binding mode results can be compared directly to alanine scanning mutagenesis data, which provides a detailed validation for a complex model. Unfortunately alanine scanning experiments are available only in a few cases, and one has to rely on binding free energies for validation in most cases.

### 2.2 Free Energy Calculations

Free energy calculations can contribute to design problems in two ways: validation of complex models as alluded above and prediction of free energy changes due to mutations. Binding constants of ligands are routinely available for most complexes and thus provide a standard test for a complex model. Several methods can be used for this purpose, from docking and scoring [4], [5] to molecular mechanics with Poisson-Boltzmann surface area (MM-PBSA) [26] and free energy calculations based on MD simulations [27], [28], [29], [30]. For a method to be useful for testing purposes, it should be able to predict binding affinities accurately. Otherwise any discrepancy with the experimental binding constant cannot necessarily be attributed to incorrect modeling. The docking and scoring methods are very fast but their accuracy for binding affinities is too poor to consider them for validation [1], [31], [32]. Similarly MM-PBSA provides a high-throughput method which has somewhat better accuracy for binding affinities, but it is still not sufficiently accurate for testing [33], [34]. Only free energy calculations based on MD have the potential to satisfy the desired level of accuracy [35], [36], [37]. The MD-based methods can be classified into two groups: i) path-independent alchemical transformation methods where the ligand is destroyed in the binding pocket while it is created in bulk; and ii) path-dependent PMF methods, where the ligand is moved from the binding pocket to bulk using biasing potentials [27], [28], [29], [30]. Alchemical methods are computationally cheaper and easier to use but their accuracy is compromised for larger, charged peptide ligands [35], which leaves the PMF method as the only choice at present for peptides.

The PMF provides the free energy profile of a ligand along a chosen reaction coordinate. The binding constant *K*_*eq*_ (inverse of the dissociation constant *K*_*D*_) of a ligand is obtained from the integration of the PMF, which is related to the standard binding free energy via *G*_*b*_ = −* kT* ln(K_*eq*_C_0_), where C_0_ is the standard concentration of 1 M. Umbrella sampling MD simulations is the most common method used in PMF calculations. The problem with sampling at high-energy positions is overcome by introducing harmonic biasing potentials along the reaction coordinate [20], [21]. The sampled coordinates of the ligand are unbiased and combined using the weighted histogram analysis method [38]. Applicability of the PMF method to peptide ligands was first shown for binding of charybdotoxin to a KcsA potassium channel mimic, where the binding free energy was calculated within chemical accuracy [39]. Since then, the PMF method has been used in several computational studies of toxin binding to ion channels (see [2], [3], [40] for reviews). Chemical accuracy was achieved in all cases, provided that a validated complex structure was employed and the PMF was calculated properly. An alternative method that has become popular in recent years due to its simplicity is to use Jarzynski's equation in steered MD simulations [41]. However, this method suffers from sampling problems and cannot provide the desired chemical accuracy for affinities [42].

Binding mode of a complex structure gives important clues on how to improve affinity and/or selectivity of a peptide ligand. By calculating the free energy change due to each suggested mutation, one can predict which one will be the most effective. Again chemical accuracy is essential in such calculations to retain predictive power, which is provided only by the MD-based methods. The two most common methods used for this purpose are free energy perturbation (FEP) and thermodynamic integration (TI) [20], [21]. In both methods, one introduces a hybrid Hamiltonian, *H*(λ) = (1 − λ)*H*_0_ + λ*H*_1_, where *H*_0_ represents the Hamiltonian in the initial state (wild-type ligand) and *H*_1_ in the final state (mutant ligand). The alchemical transformation is performed by changing the parameter λ from 0 to 1 in small steps, which ensures that the change in the free energy in each step is small enough to enable sufficient sampling of the system. In the FEP method, the interval [0, 1] is divided into *n* subintervals, and for each subinterval the free energy difference Δ*G*_*i*_ is calculate from the ensemble average. The free energy difference between the initial and final states is obtained from the sum of all Δ*G*_*i*_. In the TI method, the ensemble average of the derivative ∂* H*(λ)/∂λ is obtained at several λ values, and the free energy difference is calculated from the integral of this quantity from 0 to 1.

**Fig. 1:**
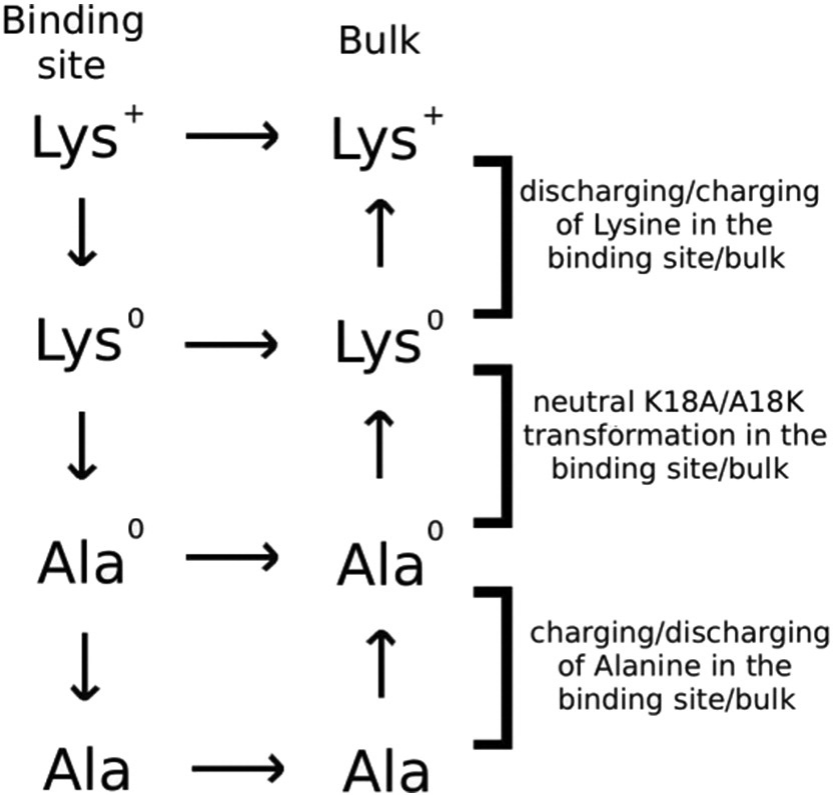
Example of a thermodynamic cycle used in free energy calculations. The superscript 0 denotes a residue with no charges on the side chain atoms. Reverse transformation is performed simultaneously in bulk to preserve the charge neutrality of the system during the FEP-MD simulations.

Charged residues have the strongest interactions, hence mutation of a neutral residue to a charged one for improving affinity (or vice versa for improving selectivity) is a common situation. This is a challenging problem that has been resolved only recently. FEP/TI calculations for mutations are usually performed separately in the binding site and bulk. This causes problems for charge mutations because the system needs to be kept neutral and also errors arise when solvation energies are calculated in different systems. In fact, such errors can be avoided by performing the binding site and bulk calculations simultaneously in the same system. That is, while a charged residue on the peptide is mutated to a neutral one in the binding site, the reverse transformation is applied simultaneously to the mutant peptide in bulk, which is well separated from the binding pocket. It is also necessary to separate the Coulomb and Lennard-Jones interactions to avoid stability and convergence problems. This can be achieved by introducing residues with uncharged side chains (denoted with a superscript 0) as intermediate steps. For example, the free energy change due to a Lys to Ala (K → A) mutation can be expressed as ΔΔ*G*_*b*_ = ΔΔ*G*(K → K^0^) + ΔΔ*G* (K^0^ → A^0^) + ΔΔ*G* (A^0^ → A). The thermodynamic cycle that combines these procedures in the FEP/TI calculations is illustrated in Fig. 1. Each of the contributions to the free energy difference can be calculated using the FEP or TI methods. The viability of this method for accurate calculation of the free energy change associated with charge mutations was shown for the K18A mutation in ShK in complex with Kv1.3, which will be discussed below [43]. The binding free energy differences obtained from the FEP/TI results were in good agreement with both the PMF and experimental results, demonstrating the feasibility and accuracy of this approach for calculation of free energy changes due to charge mutations [43].

## 3 Experimental Methods

There are many biophysical and biochemical techniques used for affinity measurements and aggregation studies of biologics. In the following, we briefly discuss some of the widely used methods.

### 3.1 Methods for Affinity Measurements

Affinity measurements involve detecting the equilibrium dissociation constant (*K*_*D*_ = *k*_*off*_/*k*_*on*_) of proteins using a variety of biophysical or biochemical techniques. The binding affinity is related to *K*_*D*_ inversely, thus a lower *K*_*D*_ value means a higher affinity. Using bioassays in measurement of the binding kinetics is among the most common methods. However, recording of the binding rate constants is not a trivial task, and sensitivity of the assay and reproducibility of the data need to be considered. Existing experimental methods to measure the *K*_*D*_ of biotherapeutics include enzyme-linked immunosorbent assay (ELISA) based methods, spectroscopy-based assays, calorimetric methods such as isothermal titration calorimetry, and a diverse range of biochemical methods.

ELISA-based methods are used for detecting and determining the amount of biomolecule under study in a quantitative manner [44]. Although there are different ELISA formats, the common points are: it is a microplate reading assay requiring an immobilized antigen or antibody (Ab) on a surface and detecting the amount of biomolecule with a spectroscopy-based technique, usually fluorescence. The antigen generally forms a complex with an antibody that is associated with an enzyme (direct assay). A secondary Ab that specifically binds to the first Ab may be used to increase the sensitivity of the method (indirect assay). In the direct assay, Ab is usually conjugated with a fluorescent dye molecule whereas in the latter the secondary Ab is labeled with a dye. In an indirect assay, the antigen is captured by an immobilized Ab prior to forming a complex with another Ab, which is often preferred due to a better sensitivity and specificity. A plate reader, for instance with fluorescence detection capability, is then used to record the signal from the tagged Ab. In some cases, the antigen can be labeled with a dye instead of Abs. Titrating for different amounts of primary or secondary Ab yields the fluorescent signal versus concentration gives information about the Ab-antigen interaction. This method is widely used, for example, to see whether or not the binding affinity is changed due to a mutation in a protein. Instead of fluorescence, other parameters such as absorbance could also be used to detect the interaction.

Another widely used method is surface plasmon resonance spectroscopy (SPR) [45]. It is based on detecting the plasmon wave that is created by the oscillating resonant electrons near a surface after a laser light induces the resonant state. To this end, the surface is coated with a metal layer (usually gold) and a protein sample is bound to this metal surface. Addition of antigen causes a change in the SPR signal, generally refractive index of the surface, enabling one to observe the intermolecular interactions including efficacy measurement of biotherapeutics. This method is commonly used for many sensor based detections as well as lab-on-a-chip applications, and there are several models available on the market. For example, antibody-antigen detection can be easily done with this method by coating the surface with the antigen. The target antibody is introduced into the system then and signal change is observed. Many antigens are available on ready to use chips, making SPR one of the high-throughput methods. If there is excessive binding compared to another protein, for example to another variant antibody with mutation, then a relative efficacy can be obtained.

Many different fluorescence properties can also be used to study molecular interactions including protein affinities. The sample is labeled with a fluorescence tag unless intrinsic tryptophan (tryp) fluorescence is used. One of the fluorescence properties (e.g. emission spectrum, fluorescence lifetime, anisotropy, energy transfer or quenching) is used to probe the interactions. A fluorimeter, lifetime instrument or a confocal microscope can be utilized for the detection of fluorescence intensity and lifetime. Depending on the interactions, a change in the fluorescence signal is expected upon binding to the target. This could be an increase or decrease in the emission maximum, a concurrent shift in the emission wavelength, a change in fluorescence lifetime or a change in polarization leading to change in anisotropy.

Flow cytometers are mainly used for cell sorting and detection but they can also be used in affinity studies. The sample flows through a steady stream created via hydrodynamic focusing in a narrow tubing and the scattered light as well as a fluorescence signal is detected from a fluorescent dye that is bound to the biomolecule of interest [46]. It is particularly useful for detecting biomolecules which bind to the cell surface, and therefore this is the preferred method to determine affinities in such systems, e.g., Abs. The number of biomolecules or cells as a function of forward or right-angle scattering are collected and can be related to the concentration and interaction of proteins.

### 3.2 Methods for Aggregation

Methods to study the structural stability and aggregation profiles of proteins can be roughly categorized into three groups [47], [48], [49]: (i) separation methods such as electrophoresis, chromatography, or centrifugation; (ii) spectroscopy based methods such as fluorescence, absorbance, light-scattering, FTIR, MS, and NMR; and (iii) microscopy based methods such as TEM, SEM, AFM, and optical. Each technique has its advantages and disadvantages and there is no single method that would be appropriate for any given system since the stability and aggregation profile of any protein is a multifaceted problem. Therefore, it is always beneficial to adapt a holistic approach and use orthogonal methods to understand the stability and aggregation issues fully. Here we will only discuss some of the widely used methods.

Intrinsic tryp fluorescence is probably the most used method for studying conformational changes in proteins, but it is also very helpful for probing protein-protein interactions [50]. Tryp fluorescence, however, becomes complicated if there is more than one tryp residue in the protein due to fluorescence being additive and also because of its solvatochromic nature. Thus, relating tryp emission to protein degradation may not be an easy task. Nevertheless, these difficulties were overcome in some studies, where tryp fluorescence was used successfully in folding and aggregation of proteins [51], [52]. Another widely used fluorescence-based technique is external dye-binding method where a protein is labeled with a fluorescent dye either covalently or by diffusion [53]. If covalent labeling approach is used, chemically different reactive moieties can be used for tagging dyes onto proteins including amine, sulfhydryl, carboxyl and glycosylation groups. These dyes generally have high quantum yields with excellent photostability. In the diffusion-based dye-binding studies, dyes are bound to either a protein or protein aggregates via diffusion of the dye molecules. A large number of dyes can be used for this purpose, and in general, these dyes have hydrophobic and aromatic structures. Consequently, they mostly bind to the hydrophobic patches on the protein surface, reporting conformational stability of the protein or intercalate inner sections of aggregates, which are usually much more hydrophobic than bulk solution. Upon binding to a hydrophobic environment, the florescence properties change radically; the emission spectra shift to a different wavelength and/or intensity is enhanced. This allows one to probe conformational changes of the protein, aggregation formation over time, or the effect of different additives on the system.

Light-scattering spectroscopy is used extensively to check aggregate formation in biotherapeutic formulations [54]. Aggregates are larger molecules compared to monomers, and hence they scatter light much more. This enables us to examine the presence of aggregates in the system and also how they are formed. Two types of scattering methods are used in experimental studies of biotherapeutics: static light scattering (SLS) and dynamic light scattering (DLS). For both methods, scattered light from a laser is detected and analyzed to reveal information on various important parameters such as the size, shape and molecular weight (MW) of molecules. SLS is based on the angle dependence of the scattered light and enables detecting absolute MW of the protein and aggregate. If the sample is heterogeneous, then it may need to be separated into constituents. Therefore, SLS systems are generally linked with a molecular separation method such as high pressure liquid chromatograph (HPLC) or flow field fractionation system. DLS uses only the right-angle detection and does not require a molecular separation method. It is used to measure hydrodynamic radius of molecules in a system. It can detect a wide range of sizes of molecules and aggregates. Many groups also apply light-scattering detection in other ways to collect information on protein aggregation, e.g., using a UV-Vis spectrophotometer or fluorimeters [55].

HPLC is the main method used in aggregation studies of proteins [49], [56]. It can be operated in different modes; size-exclusion (SEC), ion-exchange, or hydrophobic-hydrophobic interaction chromatography. SEC is a widely used method, where the sample is pushed through a tightly packed column. In the absence of any sample-column interactions, small molecules are eluted last from the column because they spend most of their time inside the column and thus they are delayed. Large molecules, i.e., protein aggregates, cannot fit in many of the cavities provided in the column, and hence they are eluted first. Other molecules come out of the column based on their sizes in between aggregates and monomers. The size of the protein of interest can be characterized by running SEC with proteins in different sizes and generating a size calibration curve. In doing so, using the same buffer, pH and flow rate for all proteins could help reduce the variations in elution times. Operating a combined system of SEC-SLS could also be helpful determining MW of eluted species from the column. The eluting peaks of the sample is observed with a UV absorbance detector, refractive index detector, or a fluorescence detector. The major limitation of the HPLC method is the time it takes to conduct an experiment. Depending on the system under study, one sample can take about 30 min. Another limitation is that very large particles cannot be detected with HPLC as they would not be able to go into the system. Column blockage is commonly observed in protein aggregation studies with HPLC. Also large particles may elute in the void volume and may not be revealed with the detector.

## 4 Applications to Biobetters

As emphasized in Methods, accurate determination of the protein-ligand complex is the most crucial step in design of biobetters whether it is for improving their affinity, selectivity or stability. Thus a proper validation of a complex structure using a variety of experimental checks is essential before proposing any mutations on a ligand. The applications discussed below are successful examples of this approach but there are many other complex structure predictions, which lack proper validation and therefore cannot be trusted for design purposes.

### 4.1 Improving Selectivity of Toxin Peptides

**Fig. 2:**
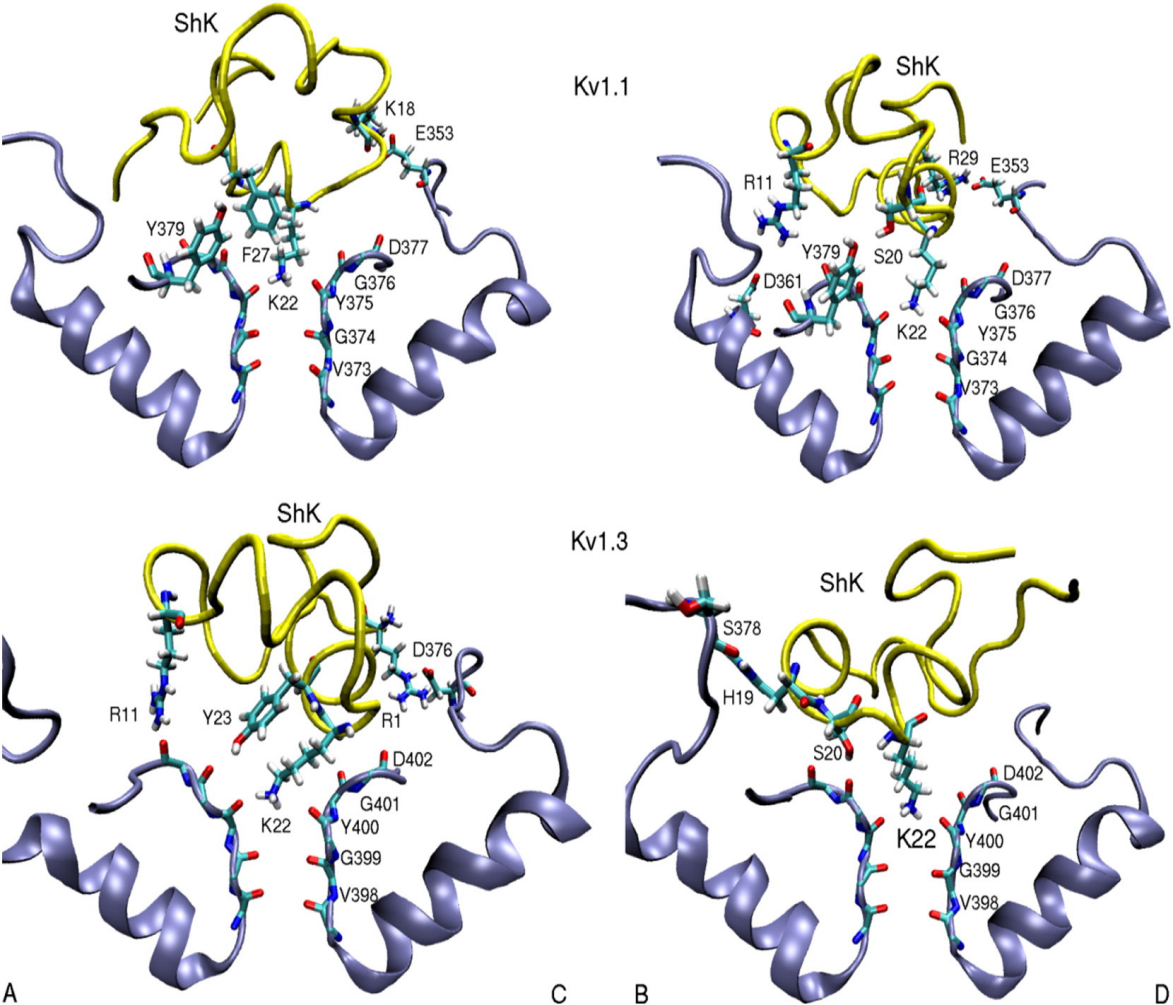
Snapshots of the Kv1.1–ShK and Kv1.3–ShK complexes. Only the strongly interacting residues involved in the binding are indicated explicitly. In order to show all the interacting pairs, two views of the complex are presented. In both cases, the pore inserting lysine (K22) blocks the pore.

Potassium channels are targeted by many toxins, which could be utilized as therapeutics in treatment of diseases caused by their dysfunction [57]. Computational studies of toxin binding to potassium channels [2], [3], [40] have been facilitated thanks to the early determination of their crystal structures [58]. Here we will focus on Kv1 channels, and in particular Kv1.3, which is an established target for the treatment of autoimmune diseases [59]. ShK toxin from sea anemone binds to Kv1.3 with a picomolar affinity, and hence is well suited for development as a therapeutic agent [59]. However, ShK has a similarly high affinity for Kv1.1 in the nervous system, and, to avoid side effects, it is essential to find analogs of ShK that are selective for Kv1.3 over Kv1.1. This is precisely the type of problem that can be addressed using the computational methods discussed here. In an initial study, the complex structures for Kv1.1–ShK and Kv1.3–ShK were constructed and validated using the available mutation data and binding free energies [60]. Comparison of the binding modes (Fig. 2) indicates some possible mutations for improving the Kv1.3/Kv1.1 selectivity, e.g., K18 and R29 on ShK make strong charge interactions with Kv1.1 but not with Kv1.3. Thus mutation of these residues to alanine should reduce its affinity for Kv1.1 without affecting Kv1.3 affinity. In the next step, free energy calculations were performed for the K18A and R29A mutations [43]. The latter changed the binding mode and was not useful but K18A was predicted to improve the Kv1.3/Kv1.1 selectivity by more than 2 kcal/mol, which was confirmed in subsequent experiments [43].

The scorpion toxin HsTx1 has a similarly high affinity for Kv1.3 and also exhibits 700-fold selectivity for Kv1.3 over Kv1.1 [61]. HsTx1 has a more stable structure than ShK, and may offer a better alternative as a therapeutic for autoimmune diseases. A similar computational study was performed for binding of HsTx1 to Kv1 channels [62]. The complex structures were validated using the binding free energies determined from PMF calculations. Comparison of the binding modes of HsTx1 with Kv1.1 and Kv1.3 showed that R14 in HsTx1 is strongly coupled to a glutamate in Kv1.1 but has no interactions with Kv1.3. Thus, the R14A mutation could further enhance the Kv1.3/Kv1.1 selectivity of HsTx1. This was followed up by performing free energy calculations for the binding of HsTx1[R14A] to Kv1.1 and Kv1.3, and more than 2 kcal/mol gain the in Kv1.3/Kv1.1 selectivity was predicted, which was confirmed in subsequent functional assay experiments [63]. While HsTx1 is more stable than ShK against degradation by enzymes [64], oral availability is still a problem. Various means have been proposed to improve the biopharmaceutical properties of peptide drugs such as cyclization [65], replacing the disulfide bridges with cystathionine bridges [66], and using lactam bridges to stabilize helical pharmacophores [67]. Yet another avenue for obtaining stable drugs is to use star polymers with functionalized ends [68].

### 4.2 Biobetters from Monoclonal Antibodies

**Fig. 3:**
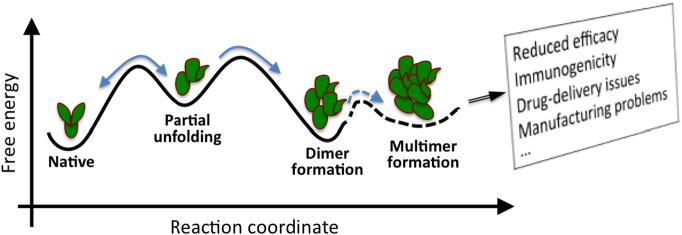
Aggregation of protein and some of the potential issues observed due to aggregation.

Monoclonal antibodies (mAbs) are the leading molecules in the biotech industry [69]. They have great pharmaceutical significance thanks to their unmatched specificity and affinity, and hundreds of mAbs are in the late stages of development [70]. Due to their large size, improving their properties poses a more challenging problem but it is still within the reach of current high performance computers. Improving the affinity/selectivity profile of a mAb follows the same script as already discussed for toxins. We will therefore focus on the aggregation problem here, which affects mAbs from development to administration. A typical path for aggregation of proteins and the ensuing consequences are shown schematically in Fig. 3. The critical step in aggregation is the partial unfolding of the protein, which exposes hydrophobic regions, followed by dimer formation that exploits the exposed regions. Thus prevention requires finding the weak points in the protein that are involved in unfolding and performing mutations at those points to prevent unfolding. If that fails, one can also try mutations that will reduce the binding affinity of another monomer.

Thus the first step in a computational study of protein aggregation is to find the partially unfolded conformations. Most of the existing approaches for predicting aggregation-prone regions of proteins are based on bioinformatics methods that search for hydrophobic regions in the amino acid sequence and use static protein structures [71]. While this approach has had some success [72], a comprehensive understanding of aggregation requires a dynamic method that will help to find the conformations leading to the dimer formation. MD simulations provide the best method for studying conformational changes is proteins but the partial unfolding of a protein is a rare process and it could take a very long time to observe. This can be overcome by performing MD simulations at higher temperatures, which will speed up unfolding of the protein [73]. Once the dominant unfolded conformer is identified, its complex structures with itself and the room temperature structure can be constructed using docking and MD. The binding free energies of the two complexes can be calculated to reveal which one is more likely to initiate aggregation. The last step is to perform mutations that will either prevent unfolding of the protein (which is expected to be harder to achieve) or reduce the binding affinity in the most stable dimer structure. The latter is similar to solving the selectivity problem for toxin peptides and follows an identical recipe. Because of the large size of mAbs, a proof of concept study was first performed for lysozyme, which does not aggregate, and its D67H mutant, which aggregates (D. Patel and S. Kuyucak, unpublished). Unfolding of the mutant lysozyme was indeed observed in high temperature MD simulations and this structure was shown to form a stable dimer with itself. The wild type lysozyme did not unfold during the same high temperature MD simulations, confirming the robustness of this approach for studies of unfolding in other proteins. In particular, application of this method to mAbs is likely to deliver novel ways to prevent their aggregation.

## 5 Summary and Outlook

Thanks to the continuing increase in computing power and developments in computational methods, we now have the ability to determine the structure of protein–ligand complexes and their binding free energies accurately. Such methods will be very useful in rational drug design in general and will facilitate development of biobetters from existing peptide and protein- based drugs. The possibility of constructing accurate complex models means that one can make rational choices for mutations to improve the affinity/selectivity profile or stability of a peptide drug lead. The effect of the chosen mutations on the binding free energy of a ligand can be determined from free energy calculations, which will minimize the experimental efforts. Although we have used peptide toxins targeting potassium channels for illustration purposes, the computational methods described here are quite general and can be applied to any receptor–ligand system, as long as their individual structures are available. In particular, developing biobetters from mAbs will greatly benefit from the computation-driven approach espoused here.
